# Correlation study on serum miR-222-3p and glucose and lipid metabolism in patients with polycystic ovary syndrome

**DOI:** 10.1186/s12905-022-01912-w

**Published:** 2022-10-01

**Authors:** Qin Wang, Chuanxiang Fang, Ying Zhao, Zhaoxia Liu

**Affiliations:** Department of Reproductive Medicine, The First Affiliated Hospital of Gannan Medical University, 23 Qingnian Road, Zhanggong District, Ganzhou, 341000 China

**Keywords:** Polycystic ovary syndrome, Overweight, miR-222-3p, Glucose and lipid metabolism, Receiver operating characteristic curve, PGC-1α, Diabetes, Cardiovascular disease

## Abstract

**Objective:**

microRNAs (miRNAs) play pivotal roles in polycystic ovary syndrome (PCOS), an endocrine and metabolic disorder that commonly occurs in women of childbearing age. This paper aimed to measure miR-222-3p expression in sera of PCOS patients and to explore its clinical value on PCOS diagnosis and prediction of diabetic and cardiovascular complications.

**Methods:**

Totally 111 PCOS patients and 94 healthy people were recruited and assigned to the overweight (ow) group and non-overweight (non-ow) group, followed by determination of serum miR-222-3p expression. The diagnostic efficiency of miR-222-3p on PCOS ow and non-ow patients was analyzed. Correlations between miR-222-3p and glycolipid metabolic indicators and diabetic and cardiovascular complications in PCOS were analyzed. The downstream target of miR-222-3p was predicted and their binding relationship was verified. The correlation between PGC-1α and miR-222-3p was analyzed.

**Results:**

miR-222-3p was highly-expressed in PCOS patients (*p* < 0.001), in especially PCOS ow patients. The area under the curve (AUC) of miR-222-3p diagnosing PCOS non-ow patients was 0.9474 and cut-off value was 1.290 (89.06% sensitivity, 98.11% specificity), indicating that non-ow people with serum miR-222-3p > 1.290 could basically be diagnosed with PCOS. AUC of miR-222-3p diagnosing PCOS ow patients was 0.9647 and cut-off value was 2.425 (85.11% sensitivity, 100% specificity), suggesting that ow people with serum miR-222-3p > 2.425 could basically be diagnosed with PCOS. miR-222-3p was positively-correlated with fasting plasma glucose (FPG), fasting insulin (FINS), homeostatic model assessment–insulin resistance (HOMA-IR), and low-density lipoprotein cholesterol (LDL-C) and negatively-correlated with high-density lipoprotein cholesterol (HDL-C). miR-222-3p was independently-correlated with diabetic and cardiovascular complications in PCOS (*p* < 0.05). High expression of miR-222-3p predicted high risks of diabetic and cardiovascular complications in PCOS. miR-222-3p targeted PGC-1α and was negatively-correlated with PGC-1α (r = − 0.2851, *p* = 0.0224; r = − 0.3151, *p* = 0.0310).

**Conclusion:**

High expression of miR-222-3p assisted PCOS diagnosis and predicted increased risks of diabetic and cardiovascular complications. miR-222-3p targeted PGC-1α and was negatively-correlated with PGC-1α.

**Supplementary Information:**

The online version contains supplementary material available at 10.1186/s12905-022-01912-w.

## Introduction

Polycystic ovary syndrome (PCOS), by definition, refers to a common endocrine disorder with heterogeneous clinical features of polycystic ovarian changes, hyperandrogenemia, and ovulatory dysfunction, which is often paired with metabolic disorders including insulin resistance (IR), diabetes, obesity, and hyperlipidemia, and patients are prone to late complications such as cardiovascular diseases (CVD) and carcinogenesis of endometrial [1–3]. PCOS can increase the risk of maternal, fetal, and neonatal complications. Pregnancy-induced hypertension syndrome, preeclampsia, gestational diabetes, spontaneous preterm birth, and increased necessity for cesarean section are the most common maternal problems; with regard to fetal outcomes, PCOS is also associated with increased neonatal incidences, premature birth, fetal growth restriction, changes in birth weight, and transfer to the neonatal intensive care unit [4]. The disorders of glucose and lipid metabolism are usually embodied as abnormal blood glucose levels, dyslipidemia, nonalcoholic fatty liver disease, weight gain, hypertension, and atherosclerotic cardio-cerebrovascular diseases [5]. The incidence of metabolic disorders in PCOS patients accounts for 18.9% in China [6]. Women suffering from PCOS often manifest with intrinsic IR [7] and enhanced cardiovascular risks [8, 9]. Hence, early diagnosis of PCOS is of clinical significance to the prevention and treatment of metabolic and cardiovascular conditions.

microRNAs (miRNAs) are small endogenous and single-stranded non-coding RNAs with a length of 19–25 nucleotides that downregulate gene expression at a post-transcriptional level [10]. miRNAs are implicated in PCOS pathogenesis [11] and are differentially expressed in PCOS patients and normal women, which is not unrelated to sex hormones and metabolism [12, 13]. miR-222 is notably up-regulated in sera and tissues of PCOS patients [12, 14], indicative of a close association with PCOS etiology. Increased miR-222-3p expression in sera of diabetic patients has a potential association with IR development [15]. Moreover, overexpression of miR-222-3p leads to a significant rise in triglyceride (TG) in hepatocytes [16]. However, we are ignorant of the clinical diagnostic value of serum miR-222-3p on PCOS and the correlation between miR-222-3p and glucose and lipid metabolism.

Peroxisome proliferator-activated receptor-γ coactivator-1α (PGC-1α) is the common target of miR-19b-3p, miR-222-3p, and miR-221-3p, which are crucial miRNAs in CVD and are able to modulate energy metabolism [17]. According to the research of Ying Liu et al., PGC-1α shows weak expression in PCOS patients, especially in PCOS obese patients [18]. In addition, PGC-1α is engaged in glucose and lipid metabolism in patients with type 2 diabetes [19]. Dehydroepiandrosterone can impede high-fat-induced hepatic glucose and lipid metabolic disorder and IR by activating the AMPK-PGC-1α-NRF-1 pathway [20], yet whether PGC-1α is involved in glucose and lipid metabolism in PCOS patients remains unclear. This study inquired into the correlation between miR-222-3p and glucose and lipid metabolism in PCOS patients, with the expectation of offering references for the metabolic disorders in PCOS patients so as to implement effective management and prevention of PCOS-related metabolic diseases and late complications such as cardiovascular complications.

## Materials and methods

### Ethics statement

This study was initiated under the approval of the Ethics Committee of The First Affiliated Hospital of Gannan Medical University (Approval number: LLSC-2021120202). Each participant was informed of this study objective and provided written informed consent. All methods were performed following the Declaration of Helsinki.

### Study subjects

The sample size was estimated beforehand using Gpower software, which gave the total sample size of ≥ 112 when effect size d = 0.4 (maximum value recommended by the system), α = 0.05 for a statistical power of 1 − β = 0.95, and *p* value obtained by two-sided tests with 4 groups (Additional file 1: Fig. S1). Female PCOS patients treated in The First Affiliated Hospital of Gannan Medical University from June 2019 to June 2021 were registered under the PCOS diagnosis criteria revised by Rotterdam consensus [21], including 64 PCOS non-overweight patients (PCOS non-ow group) who were complicated with diabetes mellitus (DM) (25 patients) and CVD (24 patients), and 47 PCOS overweight patients (PCOS ow group) who were complicated with DM (32 patients) and CVD (33 patients). At the same time, 94 healthy physical examinees, including 53 non-overweight people (control non-ow group) and 41 overweight people (control ow group) were registered as controls. Gpower estimation indicated the effect size d of 0.765 and the statistical power of > 0.8 (α = 0.05, total sample size = 205) using the equation: effect size d = mean difference/mean standard deviation (Additional file 2: Fig. S2), indicating that sample size was statistically significant.

### Inclusion criteria

PCOS patients were required to take no drugs that would affect hormones, blood glucose, and blood lipids 1 month before treatment and were diagnosed in line with the diagnostic criteria recommended by the European Society for Human Reproduction and Embryology and the American Society for Reproductive Medicine at the Rotterdam Conference in 2003 [21], which means compliance with any 2 of the following conditions: (1) oligo-ovulation or anovulation; (2) clinical or biochemical manifestations of hyperandrogenism; (3) multiple ovarian follicular cysts (unilateral ovary with ≥ 12 ovarian follicles with a diameter of 2–9 mm) and/or increased ovarian volume that was detected by ultrasonic examination, and patients with other diseases that could possibly induce hyperandrogenemia (such as hyperprolactinemia, thyroid disease, congenital adrenal hyperplasia, Cushing's syndrome, androgen-secreting tumor, and application of exogenous androgen) were excluded.

Obesity criteria: by reference to the Asia–Pacific regional guidelines proposed by the World Health Organization (WHO) and International Obesity Task Force in 2000 [obesity: body mass index (BMI) ≥ 25] [22].

Diagnostic criteria for DM were in conformity with the WHO’s 2006 diagnostic criteria for DM: fasting plasma glucose (FPG) ≥ 7.0 mmol/L; and/or blood glucose ≥ 11.1 mmol/L 2 h after sugar loading test.

Diagnostic criteria for CVD: occurrence and attack of CVD (including hypertension and hyperlipidemia); or no typical CVD symptoms, but electrocardiogram or echocardiogram indicating abnormal heart disease.

### Exclusion criteria

Older female patients (aged ≥ 35 years) associated with other endocrine diseases and hypoovarianism and a history of ovarian or (and) pelvic endometriosis were excluded. Patients with unexplained low oocyte retrieval rates, abnormal oocyte morphology, low fertilization rate, and abnormal embryo morphology were excluded.

### Detection of clinicopathological characteristics

The following information about each subject was recorded after enrollment: age, BMI, and sociodemographic characteristics (level of education, occupation, and annual income). BMI was estimated and recorded by the same physician with the same measuring instruments. The blood samples were collected on the 2nd to 3rd day of the menstrual cycle, and sex hormones including follicle-stimulating hormone (FSH), luteinizing hormone (LH), prolactin (PRL), estradiol (E2), and testosterone (T) were detected by immunochemiluminescence. The blood lipids including total cholesterol (TC), TG, low-density lipoprotein cholesterol (LDL-C), and high-density lipoprotein cholesterol (HDL-C) were analyzed using the Hitachi 7600 automatic analyzer. Fasting insulin (FINS) was detected by immunochemiluminescence and HbAlc was detected by high-pressure liquid chromatography (HPLC). Homeostatic model assessment–insulin resistance (HOMA-IR) = FPG (mmol/L) × FINS (mIU/L)/22.5. All the kits used were bought from Nanjing Xinfan biology (Nanjing, China).

### Reverse transcription-quantitative polymerase chain reaction (RT-qPCR)

The total RNA was extracted from the peripheral blood serum using TRIzol kit (Invitrogen, Carlsbad, CA, USA) and inversely transcribed into complementary DNA (cDNA) using PrimeScriptRT kit (TaKaRa, Otsu, Shiga, Japan). RT-qPCR was subsequently conducted using SYBR®PremiexExTaq™ (TaKaRa) with U6 and GAPDH as internal controls. The experiments were repeated 3 times on each sample and relative expression levels were computed using the 2^−ΔΔCt^ method. Primer sequences are demonstrated in Table 1.

**Table 1:** Primer sequences

Gene	Forward 5′–3′	Reverse 5′–3′
miR-222-3p	5′-AGCTACATCTGGCTACTGGGT-3′	5′-GCGAGCACAGAATTAATACGAC-3′
U6	5′-CTCGCTTCGGCAGCACA-3′	5′-AACGCTTCACGAATTTGCGT-3′
PGC-1α	5′-ACAGCAGCAGAGACAAATGCACC-3′	5′-TGCAGTTCCAGAGAGTTCCACACT-3′
GAPDH	5′-ATCACCATCTTCCAGGAGGGA-3′	5′-CCTTCTCCATGGTGGTGAAGAC-3′

*miR* microRNA, *PGC-1α* peroxisome proliferator-activated receptor-γ coactivator-1α, *GAPDH* glyceraldehyde-3-phosphate dehydrogenase

### Dual-luciferase reporter assay

The binding site of PGC-1α and miR-222-3p was predicted as UACAUCUG on the online website miRDB (http://mirdb.org/mirdb/index.html). Based on the prediction, the mutant (MUT) sequences and wild-type (WT) sequences of the binding site of PGC-1α and miR-222-3p were designed and cloned separately to the luciferase vector pGL3 (Promega, Madison, WI, USA) to construct PGC-1α-WT and PGC-1α-MUT luciferase plasmids. The plasmids were subsequently delivered into HEK293T cells together with miR-222-3p mimic or mimic NC for 48 h, followed by measurement of luciferase activity.

### Statistical analysis

Data analysis and plotting were undertaken using SPSS 21.0 statistical software (IBM Corp., Armonk, NY, USA) and GraphPad Prism 8.1 software (GraphPad Software Inc., San Diego, CA, USA). Shapiro–Wilk test was utilized to examine normal distribution. Measurement data complied with normal distribution were expressed as mean ± standard deviation. One-way analysis of variance (ANOVA) was adopted to analyze multi-group data, and Tukey's test was applied following ANOVA. The diagnostic efficiency of indexes was evaluated using the receiver operating characteristic (ROC) curve and the cut-off value was calculated. The influencing factors on the outcomes of DM or CVD were assessed using binary logistic regression. Independent variables were screened out using an Enter method. The *p* value was obtained with a two-tailed test. The value of *p* < 0.05 was suggestive of statistical significance.

## Results

### Comparison of clinical parameters between PCOS patients and healthy people

Totally 111 PCOS patients and 94 healthy people were recruited for this study. PCOS patients were divided into the PCOS non-ow group (N = 64) and the PCOS ow group (N = 47) following the Asia–Pacific regional guidelines proposed by the WHO and International Obesity Task Force in 2000 (obesity: BMI ≥ 25), while healthy people were allocated in the Control non-ow group (N = 53) and the Control ow group (N = 41). Sociodemographic characteristics were listed in Table 2, and no apparent difference was found in the level of education, occupation, and annual income for women born in the same period. After comparing the clinical baseline characteristics and glucose and lipid metabolism-related parameters, we observed significant differences in FPG, FINS, HOMA-IR, TG, and HDL-C between PCOS patients and healthy people, differences in BMI, FINS, HOMA-IR, TG, and HDL-C between the PCOS non-ow group and the PCOS ow group, and differences in FPG, FINS, HOMA-IR, TG, and HDL-C between the PCOS ow group and the Control ow group (all *p* < 0.5, Table 2).

**Table 2:** Clinical characteristics and sociodemographic characteristics of PCOS patients and healthy people

	PCOS		Control	
	Non-ow	ow	Non-ow	ow
	(N = 64)	(N = 47)	(N = 53)	(N = 41)
Age	27.67 ± 1.30	28.37 ± 2.09	27.79 ± 2.24	28.28 ± 1.77
Level of education [number of people (%)]				
Some high school	15 (23.44%)	14 (29.79%)	18 (33.96%)	13 (31.71%)
Completed high school	32 (50.00%)	24 (51.06%)	29 (54.72%)	20 (48.78%)
University	17 (26.56%)	9 (19.15%)	6 (11.32%)	8 (19.51%)
Occupation [number of people (%)]				
State employees	16 (25.00%)	13 (27.66%)	15 (28.30%)	16 (39.02%)
Enterprise employees	25 (39.06%)	17 (36.17%)	24 (45.28%)	19 (46.34%)
Freelancers	18 (28.13%)	15 (31.91%)	12 (22.64%)	5 (12.20%)
Students	5 (7.81%)	2 (4.26%)	2 (3.77%)	1 (2.44%)
Annual income [number of people (%)]				
0–50 thousand	31 (48.44%)	24 (51.06%)	30 (56.60%)	22 (53.66%)
50–100 thousand	24 (37.50%)	18 (38.30%)	17 (32.08%)	17 (41.46%)
> 100 thousand	9 (14.06%)	5 (10.64%)	6 (11.32%)	2 (4.88%)
BMI	21.41 ± 2.09	28.03 ± 1.92^c^	21.89 ± 1.58	27.63 ± 1.37
FPG (mmol /L)	5.56 ± 0.57^a^	5.56 ± 0.36^b^	5.26 ± 0.29	4.65 ± 0.51
HbA1c (%)	4.92 ± 0.55	4.88 ± 0.56	4.85 ± 0.39	4.82 ± 0.75
FINS (mIU /L)	11.64 ± 3.12^a^	18.23 ± 3.78^bc^	7.55 ± 1.66	7.39 ± 0.99
HOMA-IR	2.88 ± 0.84^a^	4.52 ± 1.02^bc^	1.77 ± 0.40	1.53 ± 0.26
TG (mmol/L)	2.83 ± 0.78^a^	3.87 ± 1.25^bc^	0.75 ± 0.22	1.65 ± 0.56
TC (mmol/L)	4.52 ± 0.67	4.54 ± 0.63	4.24 ± 0.41	4.34 ± 0.68
LDL-C (mmol/L)	3.10 ± 0.49	3.12 ± 0.44	2.93 ± 0.39	3.00 ± 0.33
HDL-C (mmol/L)	0.90 ± 0.29^a^	0.44 ± 0.21^bc^	2.27 ± 0.61	1.56 ± 0.21
LH (IU/L)	8.31 ± 2.11^a^	8.01 ± 1.86^bc^	5.09 ± 1.19	3.47 ± 0.81
FSH (U/L)	6.08 ± 0.97	5.81 ± 1.31^b^	6.40 ± 1.22	5.47 ± 0.87
T (ng/dL)	32.34 ± 8.30^a^	41.71 ± 12.92^bc^	22.20 ± 4.57	22.51 ± 3.49
SHBG (nmol/L)	46.77 ± 12.11^a^	28.06 ± 8.63^bc^	60.07 ± 12.197	39.56 ± 9.72
PRL (mIU/L)	437.21 ± 191.77^a^	415.07 ± 176.41	335.68 ± 109.22	375.19 ± 121.49
E2 (pg/mL)	57.79 ± 11.43^a^	47.66 ± 10.89^bc^	25.18 ± 5.55	35.45 ± 11.25

*PCOS* polycystic ovary syndrome, *ow* overweight, *BMI* body mass index, *FPG* fasting plasma glucose, *FINS* fasting insulin, *HOMA-IR* homeostatic model assessment–insulin resistance, *TG* triglyceride, *TC* total cholesterol, *LDL-C* low-density lipoprotein cholesterol, *HDL-C* high-density lipoprotein cholesterol, *LH* luteinizing hormone, *FSH* follicle-stimulating hormone, *T* testosterone, *SHBG* sex hormone-binding globulin, *PRL* prolactin, *E2* estradiol

^a^PCOS Non-ow vs. Control Non-ow, *p* < 0.05

^b^PCOS ow vs. Control ow, *p* < 0.05

^c^PCOS ow vs. PCOS Non-ow, *p* < 0.05

The levels of sex hormones were different between PCOS patients and healthy people. Significant differences in LH, T, sex hormone-binding globulin (SHBG), PRL, and E2 between the PCOS non-ow group and the Control non-ow group, differences in LH, T, SHBG, and E2 between the PCOS non-ow group and the PCOS ow group, and differences in LH, FSH, T, SHBG, and E2 between the Control ow group and the PCOS ow group were observed (all *p* < 0.5, Table 2).

### miR-222-3p was highly-expressed in serum of PCOS patients and beneficial to PCOS diagnosis

**Fig. 1:**
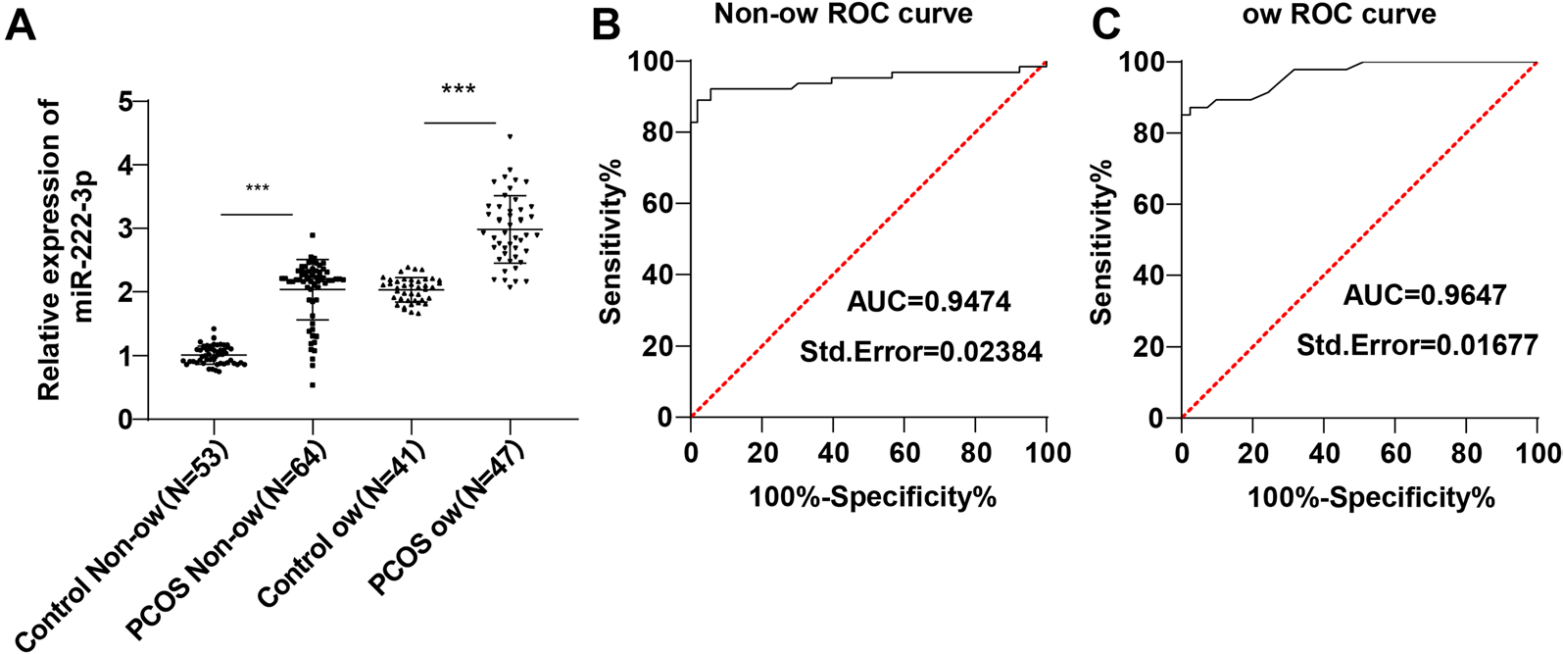
miR-222-3p was highly-expressed in sera of PCOS patients and beneficial to PCOS diagnosis. **A** expression of miR-222-3p determined by RT-qPCR; diagnostic efficiency of miR-222-3p on PCOS non-ow patients (**B**) and PCOS ow patients (**C**) analyzed using ROC curve. Multi-group comparisons in panel A were analyzed using the one-way ANOVA, and Tukey's multiple comparisons test was carried out following ANOVA. ***p < 0.001

RT-qPCR was utilized to measure the expression of miR-222-3p in sera of PCOS patients and healthy people and revealed an increase in miR-222-3p expression in the PCOS non-ow group relative to the Control non-ow group, and a rise in miR-222-3p expression in the PCOS ow group relative to the Control ow group (all *p* < 0.01, Fig. 1A). The ROC curve of miR-222-3p expression distinguishing the PCOS non-ow group and the Control non-ow group illustrated that the area under the curve (AUC) was 0.9474 and the cut-off value was 1.290 (89.06% sensitivity and 98.11% specificity) (*p* < 0.0001, Fig. 1B), suggestive of the ability of miR-222-3p > 1.290 to aid the diagnosis of PCOS in non-ow patients. Meanwhile, the ROC curve of miR-222-3p expression distinguishing the PCOS ow group and the Control ow group showed AUC of 0.9647 and cut-off value of 2.425 (85.11% sensitivity and 100% specificity) (*p* < 0.0001, Fig. 1C), indicative of the ability of miR-222-3p > 2.425 to aid the diagnosis of PCOS in ow patients.

### miR-222-3p was correlated with glucose and lipid metabolism indexes in PCOS patients

To further explore the correlation between miR-222-3p and glucose and lipid metabolism in PCOS, Pearson’s co-efficient analysis was subsequently carried out. As shown in Table 3, miR-222-3p was positively-correlated with FPG, FINS, HOMA-IR, and LDL-C (*p* < 0.05), and negatively-correlated with HDL-C in the PCOS non-ow and PCOS ow groups (*p* < 0.05).

**Table 3:** Correlation of miR-222-3p and glucose and lipid metabolism indexes in PCOS patients

	PCOS non-ow			PCOS ow	
	Pearson r	P (two–tailed)		Pearson r	P (two-tailed)
FPG	0.359	0.0036	FPG	0.4187	0.0034
HbA1c	–	0.3042	HbA1c	–	0.5275
FINS	0.513	< 0.0001	FINS	0.4218	0.0031
HOMA-IR	0.5727	< 0.0001	HOMAIR	0.4881	0.0005
TG	–	0.6214	TG	− 0.4401	0.002
TC	–	0.9332	TC	–	0.096
LDL-C	0.3446	0.0053	LDL-C	0.3046	0.0374
HDL-C	− 0.4007	0.001	HDL-C	− 0.382	0.0081

*PCOS* polycystic ovary syndrome, *ow* overweight, *FPG* fasting plasma glucose, *FINS* fasting insulin, *HOMA-IR* homeostatic model assessment–insulin resistance, *TG* triglyceride, *TC* total cholesterol, *LDL-C* low-density lipoprotein cholesterol, *HDL-C* high-density lipoprotein cholesterol

### High expression of miR-222-3p served as an independent risk factor for PCOS patients with DM

PCOS is commonly associated with clinical manifestations of metabolic syndromes including IR, DM, obesity, and hyperlipidemia. The PCOS non-ow group consisted of 25 cases of diabetic complications (39.06%) while the PCOS ow group had 32 cases of diabetic complications (68.09%). Logistic regression analysis of age, BMI, FPG, HbA1c, FINS, HOMA-IR, TG, TC, LDL-C, HDL-C, LH, FSH, T, SHBG, PRL, and E2 was conducted to analyze the independent correlation between miR-222-3p and diabetic complications in PCOS patients. Firstly, the independent risk factors for PCOS with DM were screened out using the binary regression analysis with the occurrence of diabetic complications as a dependent variable and the indexes as independent variables. The results indicated that HbA1c and miR-222-3p were independent risk factors for PCOS with DM (Table 4). For the PCOS non-ow patients and PCOS ow patients, the risk for diabetic complications was increased in patients with high miR-222-3p expression relative to those with low miR-222-3p expression (OR 70.226, 95%CI 1.369–3601.660; OR 80.293, 95% CI 2.679–2406.817).

**Table 4:** Multivariate logistic regression analysis of clinical parameters in PCOS patients complicated with diabetes

	PCOS non-ow			PCOS ow	
	*P*	OR (95% CI)		*P*	OR (95% CI)
Age	0.586	0.832 (0.43–1.613)	Age	0.695	0.875 (0.448–1.707)
BMI	0.944	0.985 (0.636–1.524)	BMI	0.737	0.893 (0.46–1.734)
FPG	0.060	2.737 (0.958–7.813)	FPG	0.723	1.353 (0.254–7.196)
HbA1c	0.003	54.454 (3.92–756.427)	HbA1c	0.029	67.278 (1.53–2958.397)
FINS	0.130	50.193 (0.315–7989.454)	FINS	0.192	38.917 (0.159–9538.746)
HOMA-IR	0.203	0.000 (0–1098.638)	HOMA-IR	0.164	0.000 (0.000–771.352)
TG	0.450	1.648 (0.45–6.032)	TG	0.407	1.591 (0.531–4.767)
TC	0.690	0.757 (0.193–2.972)	TC	0.961	0.954 (0.142–6.420)
LDL-C	0.101	0.149 (0.015–1.453)	LDL-C	0.703	1.678 (0.117–23.977)
HDL-C	0.130	62.706 (0.298–13,216.063)	HDL-C	0.413	0.054 (0.000–58.552)
LH	0.162	1.510 (0.848–2.690)	LH	0.115	1.905 (0.854–4.249)
FSH	0.662	0.797 (0.287–2.212)	FSH	0.22	0.556 (0.217–1.420)
T	0.054	1.155 (0.998–1.337)	T	0.705	1.015 (0.94–1.095)
SHBG	0.376	1.033 (0.961–1.111)	SHBG	0.797	1.020 (0.879–1.183)
PRL	0.322	1.003 (0.997–1.008)	PRL	0.509	1.002 (0.995–1.010)
E2	0.530	0.975 (0.902–1.055)	E2	0.751	1.020 (0.904–1.151)
miR-222-3p	0.034	70.226 (1.369–3601.660)	miR-222-3p	0.011	80.293 (2.679–2406.817)

*PCOS* polycystic ovary syndrome, *ow* overweight, *BMI* body mass index, *FPG* fasting plasma glucose, *FINS* fasting insulin, *HOMA-IR* homeostatic model assessment–insulin resistance, *TG* triglyceride, *TC* total cholesterol, *LDL-C* low-density lipoprotein cholesterol, *HDL-C* high-density lipoprotein cholesterol, *LH* luteinizing hormone, *FSH* follicle-stimulating hormone, *T* testosterone, *SHBG* sex hormone-binding globulin, *PRL* prolactin, *E2* estradiol, *miR* microRNA

### High expression of miR-222-3p served as an independent risk factor for PCOS patients complicated with CVD

The PCOS non-ow group had 24 cases of cardiovascular complications (37.50%) while the PCOS ow group had 33 cases of cardiovascular complications (70.21%). Logistic regression analysis of age, BMI, FPG, HbA1c, FINS, HOMA-IR, TG, TC, LDL-C, HDL-C, LH, FSH, T, SHBG, PRL, and E2 was performed to analyze the independent correlation between miR-222-3p and cardiovascular complication in PCOS patients. The analytic process was the same as that of diabetic complications. The occurrence of cardiovascular complications was taken as a dependent variable. The results suggested that TC and miR-222-3p were the independent risk factors for cardiovascular complications in PCOS non-ow and PCOS ow patients (Table 5). For the PCOS non-ow and ow patients, the risk for cardiovascular complications was increased in patients with high miR-222-3p expression relative to those with low miR-222-3p expression (OR 79.390, 95% CI 3.77–1671.674; OR 45.771, 95% CI 1.234–1697.185).

**Table 5:** Multivariate logistic regression analysis of clinical parameters in PCOS patients complicated with cardiovascular disease

	PCOS non-ow			PCOS ow	
	*P*	OR (95% CI)		*P*	OR (95% CI)
Age	0.278	0.748 (0.442–1.264)	Age	0.632	1.274 (0.473–3.426)
BMI	0.473	0.884 (0.631–1.238)	BMI	0.358	0.724 (0.364–1.441)
FPG	0.405	0.145 (0.002–13.675)	FPG	0.272	3.822 (0.349–41.847)
HbA1c	0.475	1.617 (0.433–6.038)	HbA1c	0.339	3.942 (0.237–65.454)
FINS	0.215	0.223 (0.021–2.386)	FINS	0.519	7.930 (0.015–4283.911)
HOMA-IR	0.298	1.108 (0.607–2.025)	HOMA-IR	0.325	1.481 (0.678–3.236)
TG	0.469	0.711 (0.283–1.789)	TG	0.960	1.039 (0.239–4.514)
TC	0.044	3.173 (1.029–9.78)	TC	0.043	23.825 (1.099–516.505)
LDL-C	0.409	0.482 (0.085–2.724)	LDL-C	0.162	8.385 (0.426–165.198)
HDL-C	0.964	0.937 (0.055–16.031)	HDL-C	0.816	1.805 (0.012–261.406)
LH	0.090	0.74 (0.523–1.048)	LH	0.317	0.623 (0.246–1.575)
FSH	0.575	0.797 (0.36–1.764)	FSH	0.383	0.647 (0.243–1.722)
T	0.084	0.923 (0.843–1.011)	T	0.799	1.013 (0.92–1.115)
SHBG	0.198	0.961 (0.906–1.021)	SHBG	0.907	1.012 (0.834–1.227)
PRL	0.832	1.000 (0.997–1.004)	PRL	0.577	1.003 (0.994–1.011)
E2	0.859	0.995 (0.939–1.054)	E2	0.438	1.057 (0.919–1.216)
miR-222-3p	0.005	79.390 (3.77–1671.674)	miR-222-3p	0.038	45.771 (1.234–1697.185)

*PCOS* polycystic ovary syndrome, *ow* overweight, *BMI* body mass index, *FPG* fasting plasma glucose, *FINS* fasting insulin, *HOMA-IR* homeostatic model assessment–insulin resistance, *TG* triglyceride, *TC* total cholesterol, *LDL-C* low-density lipoprotein cholesterol, *HDL-C* high-density lipoprotein cholesterol, *LH* luteinizing hormone, *FSH* follicle-stimulating hormone, *T* testosterone, *SHBG* sex hormone-binding globulin, *PRL* prolactin, *E2* estradiol, *miR* microRNA

### PGC-1α was weakly-expressed in serum of PCOS patients and negatively-correlated with miR-222-3p

**Fig. 2:**
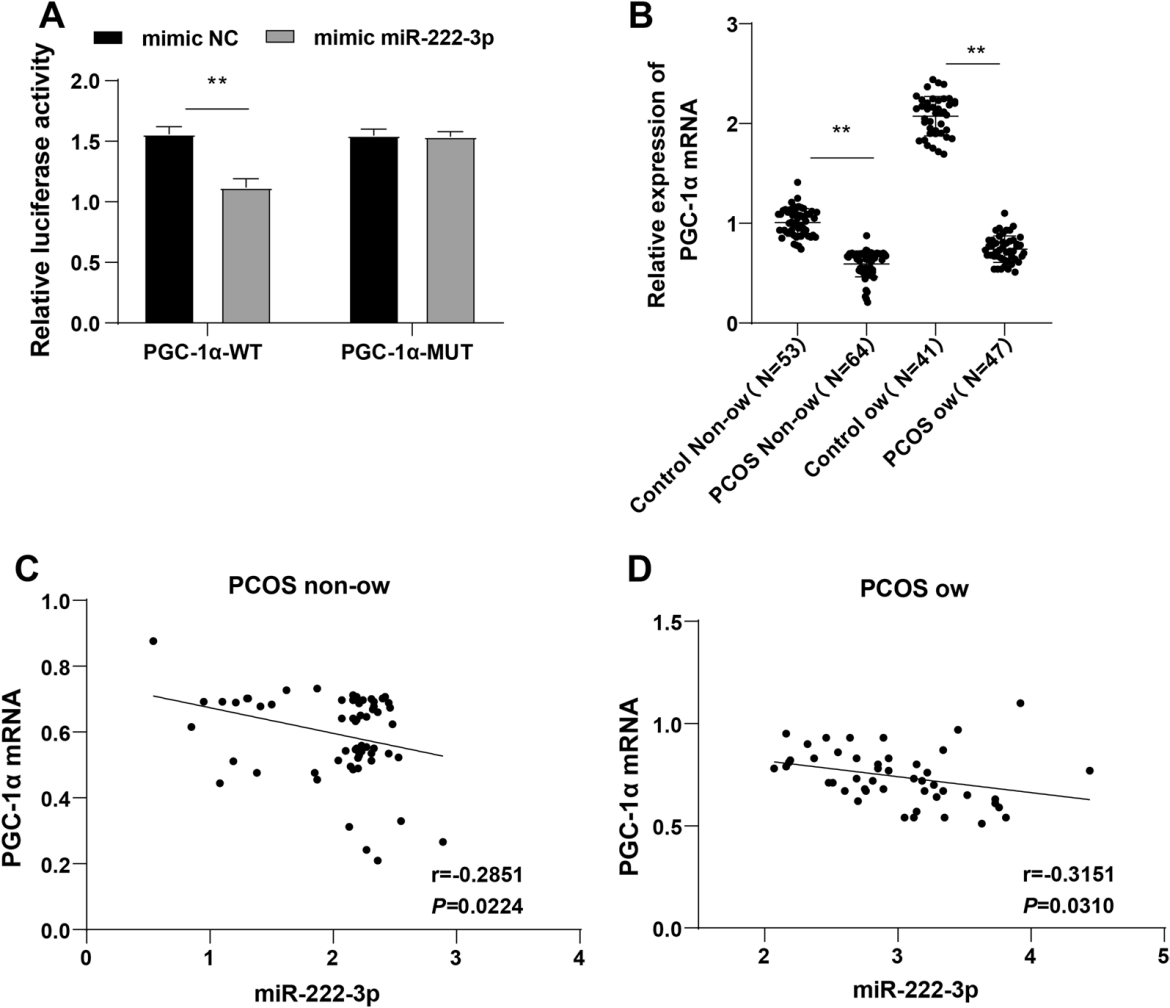
PGC-1α was weakly-expressed in sera of PCOS patients and negatively correlated with miR-222-3p. **A** binding relationship between miR-222-3p and PGC-1α verified by the dual-luciferase reporter assay; **B** expression of PGC-1α determined by RT-qPCR; **C**, **D** correlation of PGC-1α and miR-222-3p in sera of PCOS non-ow and PCOS ow patients analyzed using the Pearson’s coefficient analysis

PGC-1α is poorly-expressed in PCOS patients [18] and is involved in the modulation of glucose and lipid metabolism in patients with type 2 DM by mediating the aberrant expression of mitochondrial oxidative phosphorylation (OXPHOS) [19]. PGC-1α was confirmed as the target gene of miR-222-3p according to the predicted result of miRDB database (http://mirdb.org/mirdb/index.html), and their binding relationship was verified by the dual-luciferase reporter assay (Fig. 2A). RT-qPCR exhibited a lower expression of PGC-1α in the PCOS non-ow group than the Control non-ow group and a lower expression of PGC-1α in the PCOS ow group than the Control ow group (all *p* < 0.01, Fig. 2B). Pearson’s coefficient analysis showed that PGC-1α was weakly-expressed in sera of PCOS non-ow and ow patients and negatively-correlated with miR-222-3p (all *p* < 0.05, Fig. 2C, D). These results elicited that miR-222-3p might produce important effects on glucose and lipid metabolism in PCOS patients by targeting PGC-1α. The original data are available as additional file (see Additional file 3 for the original data for Fig. 1 and Fig. 2).

## Discussion

PCOS is an endocrine-metabolic disorder highly prevalent in women of reproductive age [23]. Glucose and lipid metabolic disorder and obesity are common accompaniments to PCOS [24]. The association between miR-222 and lipid metabolism is a known fact [25]. Moreover, miR-222 is implicated in PCOS [14]. This study investigated the correlation between miR-222-3p and glucose and lipid metabolism in PCOS patients. Our results illuminated that high expression of miR-222-3p could aid PCOS diagnosis and predict the increased risk of diabetes and CDV, and miR-222-3p targeted PGC-1α and was negatively associated with PGC-1α.

A significant rise in miR-222 expression has been observed in PCOS patients [26]. Likewise, our study revealed increased expression of miR-222-3p in PCOS ow patients and PCOS non-ow patients compared to healthy ow people and healthy non-ow people. The ROC curve demonstrated that the serum level of miR-222-3p > 1.290 could aid the diagnosis of PCOS non-ow patients while serum level of miR-222-3p > 2.425 could aid the diagnosis of PCOS ow patients. A previous finding of the diagnostic value of miR-222 on PCOS [12] is strong support to our finding that up-regulated miR-222-3p was beneficial to PCOS diagnosis.

The elevation of miR-222 expression contributes to an increase in glucose metabolism indicators in PCOS rats [27]. miR-222-3p can exert regulatory effects on lipid metabolism in atherosclerosis [28]. After measuring the glucose and lipid metabolic indicators in PCOS ow and non-ow patients, we confirmed that miR-222-3p was positively correlated with FPG, FINS, HOMA-IR, and LDL-C and negatively correlated with HDL-C.

Complications including DM and CVD are long-term consequences of PCOS [29]. In this study, we firstly identified the independent correlation between miR-222-3p and diabetic complications. HbA1c has shown beneficial aspects in screening PCOS complications [30]. In our study, HbA1c and miR-222-3p served as independent risk factors for diabetic complications in PCOS. Furthermore, a high level of TC is one of the contributors to CVD [31]. Our results indicated that TC and miR-222-3p acted as the independent risk factors for CVD in PCOS non-ow patients. The deregulation of miR-222 expression is implicated in a series of DM and CVD [32, 33]. Collectively, high expression of miR-222-3p was correlated with increased risks of diabetic and cardiovascular diseases in PCOS patients.

As reported in a previous study, PGC-1α is implicated in PCOS [34]. We then confirmed the binding relationship between PGC-1α and miR-222-3p by the dual-luciferase reporter assay. Consistent with former research [18], PGC-1α was weakly expressed in PCOS patients. miR-222-3p suppresses PGC-1α in atherosclerosis [17]. Combined with our finding that PGC-1α was negatively correlated with miR-222-3p in PCOS patients, it could be inferred that miR-222-3p might play a regulatory role in glucose and lipid metabolism in PCOS patients by targeting PGC-1α.

To sum up, miR-222-3p was highly-expressed in PCOS ow and non-ow patients, and high expression of miR-222-3p could aid the diagnosis of PCOS and severity assessment and imply increased risks of diabetic and cardiovascular complications. Meanwhile, miR-222-3p targeted PGC-1α and played an essential role in folliculogenesis. This study offered a new reference for the efficacy of miR-222-3p in PCOS diagnosis and severity evaluation and prediction of diabetic and cardiovascular complications. The limitation of this study was that the number of cases and events included and analyzed was relatively small. Future research shall aim to further clarify the diagnostic and prognostic abilities of miR-222-3p and expand the investigation into the target genes of miR-222-3p based on larger sample size and different phenotypes of PCOS to increase the credibility of the results.

## Supplementary Information

**Additional file 1: Fig. S1.** Sample size was estimated in advance using Gpower software.

**Additional file 2: Fig. S2.** Statistical power of differential expression of miR-222-3p in different groups was estimated using Gpower software.

**Additional file 3.** Original data for Fig. 1 and Fig. 2.

## Data Availability

All data generated or analysed during this study are included in this published article [and its Additional files].
