# Supplementary figures and images for: Correlation study on serum miR-222-3p and glucose and lipid metabolism in patients with polycystic ovary syndrome
Source: BMC Womens Health. 2022 Oct 1;22:398. doi: 10.1186/s12905-022-01912-w (PMC9526283; doi:10.1186/s12905-022-01912-w)

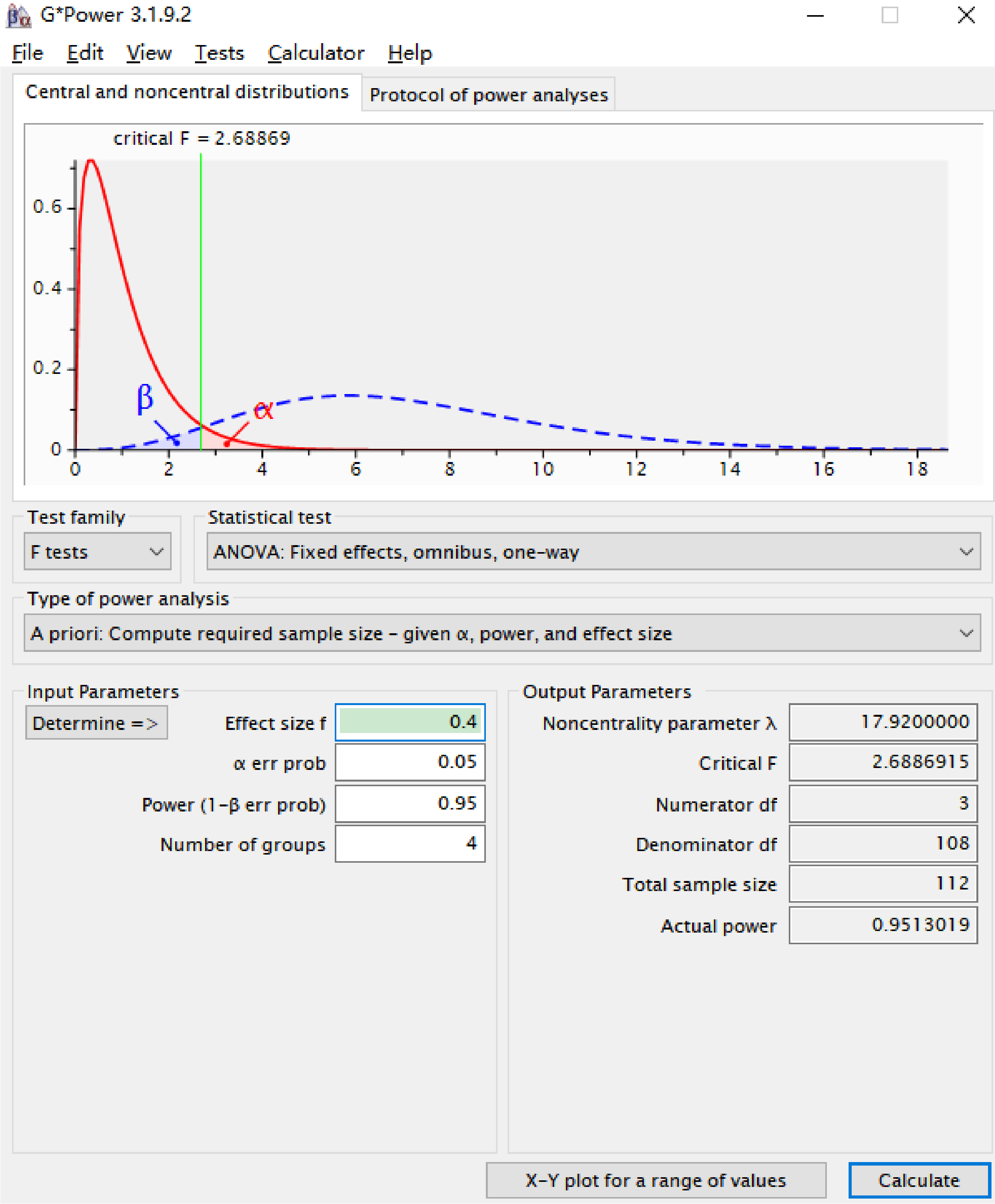

Supplement: Supplementary file 1 — Additional file 1: Fig. S1. Sample size was estimated in advance using Gpower software. [file 12905_2022_1912_MOESM1_ESM.tiff]

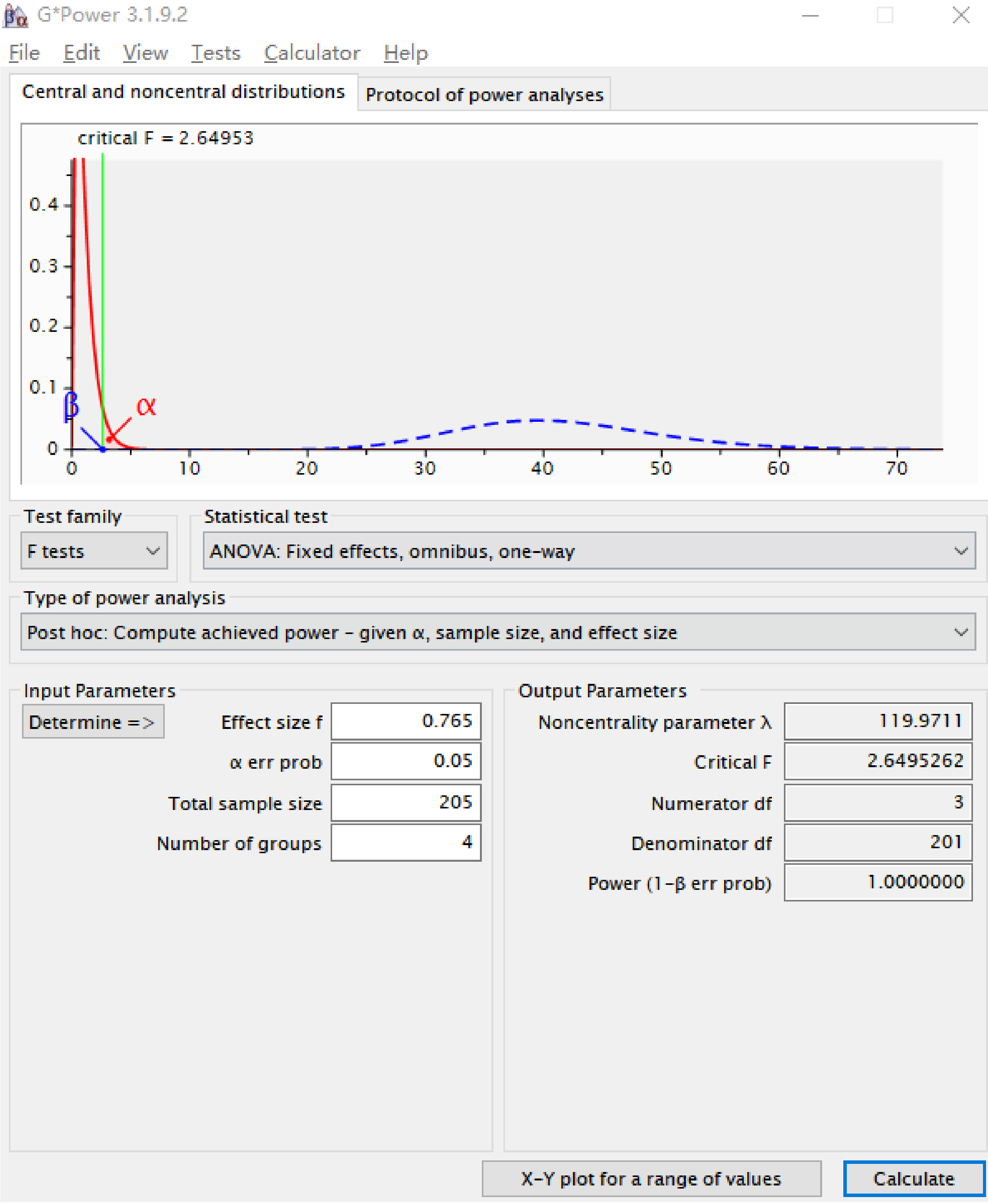

Supplement: Supplementary file 2 — Additional file 2: Fig. S2. Statistical power of differential expression of miR-222-3p in different groups was estimated using Gpower software. [file 12905_2022_1912_MOESM2_ESM.tiff]
